# Effects of Developing Primary Caregivers’ Self-Efficacy on Caring Behavior for Underweight Preschool Children in the Child Development Centers, Nakhon Si Thammarat Province

**DOI:** 10.3390/healthcare14131891

**Published:** 2026-06-29

**Authors:** Wiphada Khocharoen, Thidarat Eksirinimit, Kiatkamjorn Kusol

**Affiliations:** 1 Walailak University Medical Center Hospital, Walailak University, Nakhon Si Thammarat 80000, Thailand; momandtonnam@gmail.com; 2 Excellence Center of Community Health Promotion, School of Nursing, Walailak University, Nakhon Si Thammarat 80160, Thailand; kkiatgum@gmail.com

**Keywords:** caregiver self-efficacy, underweight preschool children, nutrition intervention

## Abstract

**Background**: Underweight in preschool children remains a significant public health concern, particularly in the community where caregivers are key contributors to child nutrition. This quasi-experimental study aimed to assess the effectiveness of a caregiver self-efficacy-based intervention on caregiving practices and nutritional outcomes. **Materials and Methods**: The study was conducted over three months at a child development center in Tha Sala District, Nakhon Si Thammarat, Thailand. The study involved 60 primary caregivers of underweight preschool children (aged 2.5–5 years). The study was conducted in Tha Sala District, Nakhon Si Thammarat Province, which was selected by simple random sampling from the five districts with the highest prevalence of underweight preschool children. Ten child development centers were randomly selected from 28 centers and randomly allocated to the experimental group (5 centers) or the control group (5 centers), with 30 participants in each group. The intervention was designed to improve caregiver self-efficacy through structured education, practical skills training, and support provided for effective behavior practice. We collected data using structured questionnaires on demographic characteristics, health status of the child, self-efficacy of the caregiver, and caregiving practices. Participant characteristics were summarized using descriptive statistics. The Wilcoxon signed-rank test was used to compare differences within groups, and the Mann–Whitney U test compared differences between groups. **Results**: After the intervention, caregivers in the experimental group had significantly better self-efficacy and caregiving practice scores compared to both baseline and control (*p* < 0.05). In addition, the experimental group had greater improvements in body weight. The findings demonstrate the effectiveness of self-efficacy-based interventions in enhancing caregiver behaviors and child nutritional status. **Conclusions**: Incorporating such interventions into community-based child health programs has the potential to increase the effectiveness of interventions and can serve as a sustainable approach to reducing childhood undernutrition in comparable settings.

## 1. Introduction

Underweight among preschool children is defined as a weight-for-age measurement below −2 standard deviations (SD) of the WHO Child Growth Standards for children under five years old [1]. According to the Department of Health guidelines, underweight is defined as a weight-for-age value below −2 standard deviations (SD) from the median of the child growth standards, whereas slightly underweight refers to a weight-for-age value between −1.5 and −2 SD from the median [2]. These conditions are commonly associated with inadequate dietary intake and insufficient consumption of energy and essential nutrients, which may adversely affect normal growth and development in children [2]. Underweight remains a pressing global public health issue, affecting an estimated 45 million children in 2022, with the greatest burden in low- and middle-income countries, especially across Africa and Asia [1,3]. In Thailand, national surveillance data indicated that about 8% of preschool children aged 2–5 years were underweighted in 2019, and this figure is projected to rise to 9.85% by 2024 [4,5,6]. In Nakhon Si Thammarat Province, the prevalence is even higher, at 11.34%, with particularly elevated rates in districts such as Bang Khan (12.52%) and Tha Sala (12.39%) [4]. These figures underscore underweight as a persistent and serious public health challenge among preschool children in this region.

The intervention program in this study was developed based on Bandura’s Self-Efficacy Theory, which suggests that individuals’ beliefs in their capabilities to perform specific behaviors influence their motivation and behavioral outcomes [7]. Previous studies have demonstrated that self-efficacy enhancement programs can improve caregivers’ confidence and caregiving practices related to child health and nutrition [8,9,10]. Furthermore, caregivers play a critical role in determining children’s nutritional status through food preparation, feeding practices, and daily care behaviors [11,12,13,14].

Although previous studies in Thailand have reported factors associated with undernutrition and nutritional caregiving practices among preschool children [12,13,14], and a local study in Tha Sala District identified inappropriate food consumption behaviors among preschool children [15], there remains limited evidence regarding theory-based interventions targeting primary caregivers of underweight preschool children in Nakhon Si Thammarat Province [4]. Therefore, this study was conducted to address this knowledge gap by evaluating the effectiveness of a self-efficacy enhancement program for primary caregivers. The findings are expected to provide evidence supporting nutritional promotion strategies for underweight preschool children and contribute to the development of community-based child nutrition programs in Thailand [2,16]. Supportive practices such as providing nutritionally adequate, energy-dense diets and maintaining consistent meal routines are tied to better nutritional outcomes [13,17,18,19]. In contrast, inappropriate feeding behaviors—such as frequent offerings of processed foods or sugary drinks, and coercive feeding methods—may decrease dietary intake and heighten the risk of malnutrition [11,20]. According to Bandura’s social cognitive theory, self-efficacy is a central determinant of behavior change, developed through mastery experiences, vicarious learning, verbal encouragement, and emotional arousal [7]. Strengthening caregiver self-efficacy may enhance their capacity to sustain healthy caregiving and feeding practices. Evidence from prior studies shows that interventions targeting self-efficacy can improve caregiver behaviors and children’s nutritional status. For instance, nutrition rehabilitation programs grounded in self-efficacy theory have produced positive changes in the nutritional status of malnourished children under five [19]. Similarly, caregiver-centered and family-based interventions have led to gains in feeding behaviors, nutrition knowledge, and weight-for-age among underweight children [11,12,16,20].

Given the high prevalence of underweight among preschool children in Nakhon Si Thammarat Province, there is a need for effective, theory-based interventions targeting caregivers. Therefore, this study developed and implemented a self-efficacy-based intervention program for primary caregivers of underweight preschool children attending child development centers. The program incorporated the four sources of self-efficacy and was adapted to the local sociocultural context. Digital communication tools, including the LINE application, were also used to provide continuous education, social support, and follow-up. The objectives of this study were to (1) examine changes in primary caregivers’ self-efficacy and caregiving practices, as well as children’s body weight and nutritional status, following participation in a self-efficacy-based intervention, and (2) compare these outcomes between the experimental and control groups.

## 2. Materials and Methods

### 2.1. Study Design

This study employed a quasi-experimental two-group pretest–posttest design. Child development centers were assigned to either the experimental or control group, and eligible caregiver–child dyads within each center were recruited accordingly. The researcher first identified the five districts with the highest prevalence of underweight preschool children among the 23 districts in Nakhon Si Thammarat Province. One district was then selected using simple random sampling, resulting in Tha Sala District being chosen as the study site. From the 28 child development centers in Tha Sala District, 10 centers were selected through simple random sampling. To minimize contamination between participants, the child development center was used as the unit of assignment. The selected centers were then randomly allocated to either the experimental group (5 centers) or the control group (5 centers). Eligible primary caregivers and their preschool children from the selected centers were subsequently recruited into their assigned study groups. The experimental group received a self-efficacy-based intervention, while the control group received routine care provided by the child development centers. The control group received routine care as regularly provided by the child development centers during the study period. Routine care included two snack meals and one lunch meal each day in accordance with the Thai School Lunch Program guidelines. The quantity and nutritional proportions of meals were standardized across all participating child development centers. In addition, teachers routinely provided general advice regarding appropriate nutrition and feeding practices to children and their primary caregivers. No structured nutritional intervention, caregiver training, or additional activities beyond the centers’ usual care practices were provided to the control group. Children aged 2 years and 6 months to 5 years were included because this age range corresponds to the preschool period covered by child development centers. Furthermore, children in this age group remain largely dependent on caregivers for feeding and nutritional care, making them suitable for evaluating the effects of the intervention on caregiver behaviors and child weight outcomes. who were enrolled in 363 child development centers across 23 districts under Subdistrict Administrative Organizations in Nakhon Si Thammarat Province, Thailand (N = 18,260) [21], and their primary caregivers who resided in the same household and were responsible for the child’s daily care.

Both the control and experimental groups received routine care provided by the child development centers. This included two snack meals and one lunch meal each day. Meals were provided in accordance with the Thai School Lunch Program guidelines, and the quantity and nutritional proportions were standardized across all participating child development centers.

### 2.2. Measures

The study sample included underweight preschool children aged 2 years 6 months to 5 years attending child development centers under Subdistrict Administrative Organizations in Tha Sala District, Nakhon Si Thammarat Province, and their primary caregivers. The sample size was calculated based on effect size estimates from a previous study examining the effects of a parental feeding behavior promotion program on nutritional outcomes among underweight preschool children [12]. Using an effect size of 0.70, a statistical power of 0.80, and a significance level of 0.05, the required sample size was calculated using G*Power version 3.1.9.7, resulting in 26 participants per group. To account for potential attrition, the sample size was increased by 30%, yielding a target sample of 34 participants per group.

The inclusion criteria were as follows: Children: (1) Aged between 2 years 6 months and 5 years; (2) Enrolled continuously in a child development center under the Subdistrict Administrative Organization in Tha Sala District; (3) Classified as underweight (weight-for-age < −2 SD) or slightly underweight (weight-for-age between −1.5 and −2 SD) according to the child growth standards of the Department of Health, Ministry of Public Health, Thailand [2]; and (4) No underlying medical conditions affecting growth or nutritional status, including thyroid disease, diabetes, thalassemia, heart disease, kidney disease, cancer, or hematologic disorders.

Participants who were unable to complete all intervention activities or who withdrew from the study after enrollment were considered attrition cases and reported as lost to follow-up. During the study period, four participants withdrew from the study and were excluded from the final analysis. However, the remaining sample size was sufficient for statistical analysis according to the calculated sample size requirements. Complete data were obtained from all participants who completed the study; therefore, no missing data imputation procedures were required.

### 2.3. Research Instruments

The research instruments consisted of intervention tools and outcome assessment tools.

Intervention tools:

1. The Nurse’s Guide to Self-Efficacy Development for Primary Caregivers of Underweight Preschool Children.
2. The Caregiver Handbook for Underweight Preschool Children.

Outcome assessment tools:

1. A structured questionnaire assessing demographic characteristics of primary caregivers and health information of preschool children.
2. A structured questionnaire assessing caregiver self-efficacy and caregiving practices related to caring for underweight preschool children.

The questionnaire on primary caregivers’ self-efficacy and caregiving behaviors for underweight preschool children consisted of two sections and was developed using a 5-point Likert scale.

The self-efficacy questionnaire comprised 22 items covering three domains: food ingredient selection (5 items; score range 5–25), food preparation (6 items; score range 6–30), and feeding care practices (11 items; score range 11–55). The total self-efficacy score ranged from 22 to 110, with higher scores indicating greater perceived self-efficacy in caring for underweight preschool children.

The caregiving behavior questionnaire consisted of 33 items in three domains: food ingredient selection (8 items; score range 8–40), food preparation (14 items; score range 14–70), and feeding care practices (11 items; score range 11–55). The total caregiving behavior score ranged from 33 to 165, with higher scores indicating more appropriate caregiving behaviors for underweight preschool children.

Mean scores for self-efficacy and caregiving behaviors, both by domain and overall, were interpreted using five levels: very low, low, moderate, high, and very high.

Content validity was evaluated using the Content Validity Index (CVI), yielding a value of 0.96, indicating excellent content validity. Internal consistency reliability was assessed using Cronbach’s alpha coefficient. The questionnaire demonstrated excellent reliability, with a Cronbach’s alpha coefficient of 0.97. In the present study, the instrument also showed excellent internal consistency reliability, with a Cronbach’s alpha coefficient of 0.97.

#### 2.3.1. Intervention Procedure

The intervention was developed based on Bandura’s Self-Efficacy Theory [7], incorporating four key sources of self-efficacy: mastery experience, vicarious experience (role modeling), verbal persuasion, and emotional support. The intervention was conducted over 12 weeks. Both the experimental and control groups completed baseline assessments of caregiver self-efficacy and caregiving practices using structured questionnaires prior to the intervention. The control group received routine care provided by the child development centers, while the experimental group participated in the self-efficacy-based intervention program.

#### 2.3.2. Week 1: Orientation and Group Session

An initial group session was conducted to introduce the study objectives, procedures, and participant rights, and to obtain informed participation. Role models were identified in collaboration with teachers from child development centers. These role models were primary caregivers who had successfully cared for underweight preschool children and met the study eligibility criteria. Group discussions focused on key caregiving domains, including food purchasing, food preparation, and feeding and childcare practices. Role models shared their experiences, practical strategies, and problem-solving approaches to support appropriate caregiving behaviors.

#### 2.3.3. Weeks 2–11: Self-Efficacy Promotion and Follow-Up Support

Educational and behavioral support activities were conducted through a combination of face-to-face and online sessions. Structured educational sessions were conducted during Weeks 2, 4, and 8, with each session lasting approximately 30–45 min. The content focused on enhancing caregiver knowledge, skills, and confidence in providing appropriate nutrition and care. The intervention was delivered via LINE by organizing caregivers into 10 separate groups (5 intervention and 5 control) based on their child development centers. This approach provided a practical and accessible follow-up tool that eliminated the need for face-to-face visits while enhancing study adherence and continuity of care. LINE is the most widely used mobile communication application in Thailand, enabling instant messaging, image sharing, and video communication. Through these groups, the researcher shared educational materials, activity reminders, and timely nutritional guidance, while caregivers shared meal photographs for continuous monitoring. Furthermore, the platform created a supportive environment for caregivers to exchange experiences and seek advice from both peers and the researcher, highlighting the value of mobile applications in community-based public health programs.

The researcher disseminated educational content to caregivers focusing on three key domains of child care behaviors: food selection, food preparation, and feeding practices for preschool children. These activities were designed to reinforce mastery experiences, promote observational learning, strengthen caregivers’ confidence through verbal encouragement, and provide emotional support, in accordance with the principles of self-efficacy theory.

#### 2.3.4. Week 12: Post-Intervention Assessment

At the end of the intervention period, post-intervention assessments were conducted using the same structured questionnaires to evaluate caregiver self-efficacy and caregiving practices. Data collection was facilitated by trained research assistants, and clarification was provided when necessary. The intervention was formally concluded after completion of all assessments.

#### 2.3.5. Anthropometric Measurement

To ensure the accuracy and consistency of anthropometric measurements, ten digital weighing scales of the same brand were calibrated and standardized before data collection and provided to each child development center for measuring children’s body weight. Weight measurements were conducted in the morning at all centers using the same procedure. Children were weighed wearing light clothing and without shoes. Body weight was recorded in kilograms to the nearest 0.1 kg. Height was measured using standard standing height measuring equipment available at each child development center in accordance with the Ministry of Public Health guidelines. Measurements were recorded in centimeters to the nearest 0.1 cm. During height measurement, children were asked to remove their shoes and stand upright with their heels against the wall, knees together, and back and head positioned against the wall. All weight and height measurements were performed by the researcher to ensure consistency across study sites.

#### 2.3.6. Outcome Assessment Tools

Anthropometric measurements of preschool children, including body weight and height. Body weight was measured using calibrated digital weighing scales in the morning while children wore light clothing and no shoes, and values were recorded to the nearest 0.1 kg. Height was measured using standard standing height measuring equipment in accordance with Ministry of Public Health guidelines and recorded to the nearest 0.1 cm.

### 2.4. Data Collection

Outcome data were collected at two time points: baseline and immediately after completion of the 12-week intervention. Measures included caregiver self-efficacy, caregiving behaviors, and children’s body weight. During the study period, four participants in each group withdrew or were lost to follow-up. Therefore, the final sample consisted of 30 caregiver–child dyads in the experimental group and 30 caregiver–child dyads in the control group.

All data were collected by trained research assistants under the supervision of the researchers to ensure accuracy and consistency. Standardized measurement procedures were applied throughout the study to minimize measurement bias and enhance data reliability.

### 2.5. Ethical Statement

This study was approved by the Institutional Review Board of Walailak University (Approval No. WUEC-25-123-01; approved on 18 April 2025) and was conducted in accordance with the ethical principles of the Declaration of Helsinki. Before enrollment, primary caregivers were provided with clear information about the study, including its purpose, procedures, potential risks and benefits, and their rights as participants. Written informed consent was obtained from all participants prior to data collection. Participation was voluntary, and caregivers were informed that they could withdraw from the study at any time without any consequences. All data were handled confidentially and used exclusively for research purposes. Personal identifiers were removed to protect participant anonymity, and all research data were securely stored and accessible only to the research team.

### 2.6. Statistical Analysis

Descriptive statistics were used to summarize the baseline characteristics of primary caregivers and preschool children. Categorical variables are presented as frequencies and percentages, whereas continuous variables are expressed as means and standard deviations. Baseline differences between the experimental and control groups were examined using the Chi-square test or Fisher’s exact test for categorical variables, and the independent samples *t*-test for continuous variables, as appropriate.

The normality of continuous variables was evaluated using the Kolmogorov–Smirnov test, along with assessment of skewness and kurtosis. The results indicated that the data deviated from normality (*p* < 0.05), and skewness and kurtosis values were outside acceptable ranges. Accordingly, non-parametric tests were used for further analyses. Within-group changes in caregiver self-efficacy, caregiving practices, and children’s body weight before and after the intervention were assessed using the Wilcoxon signed-rank test. Between-group differences in caregiver self-efficacy, caregiving practices, and changes in children’s body weight and nutritional outcomes were evaluated using the Mann–Whitney U test.

All statistical analyses were performed using IBM SPSS Statistics (version 26; IBM Corp., Armonk, NY, USA). A two-tailed *p*-value of less than 0.05 was considered statistically significant.

## 3. Results

During the study period, four participants in each group withdrew from the study or were lost to follow-up. As a result, the final sample consisted of 30 caregiver–child dyads in each group. This sample size remained above the minimum required sample size and was sufficient to maintain the statistical power of the study.

A total of 60 preschool children were included in the study, comprising 31 boys (51.67%) and 29 girls (48.33%). The largest proportion of children were aged > 4–5 years (43.33%). More than half had no dental caries (56.67%), consumed three main meals per day, and drank two cartons of milk daily (55.00%).

Regarding nutritional status, 58.33% of the children were classified as slightly underweight, while 41.67% were underweight.

No statistically significant differences were observed between the experimental and control groups in baseline characteristics, including sex, age, age group, birth order, dental health, meal frequency, milk consumption, or nutritional status (all *p* > 0.05). These findings indicate that the two groups were comparable prior to the intervention (Table 1).

### 3.1. General Information of Primary Caregivers

Among the 60 primary caregivers, 91.67% were female, and 8.33% were male. Most caregivers were aged 20–40 years (81.67%). The majority were the child’s mother or father (86.67%), were married (75.00%), and had completed secondary education (60.00%).

The mean monthly family income was 11,450.00 ± 4188.14 baht in the experimental group and 11,350.00 ± 3651.05 baht in the control group. Baseline comparisons showed no statistically significant differences between the two groups in demographic characteristics, self-efficacy scores, or caregiving behavior scores (*p* > 0.05), confirming group equivalence at baseline (Table 2).

### 3.2. Effects of the Self-Efficacy Promotion Program

#### 3.2.1. Within-Group Comparisons (Pre–Post Intervention)

Wilcoxon signed-rank tests showed that, in the experimental group, primary caregivers’ self-efficacy scores across all domains (food purchasing, food preparation, and feeding and care) and total self-efficacy scores were significantly higher after the intervention than at baseline (*p* < 0.001).

Similarly, caregiving behavior scores in food preparation and feeding and care, as well as total caregiving behavior scores, showed significant improvements following the intervention (*p* < 0.001). However, food purchasing behaviors did not change significantly (*p* > 0.05).

In addition, the mean body weight of preschool children in the experimental group increased significantly from 12.48 ± 1.65 kg at baseline to 13.73 ± 1.92 kg after the intervention (*p* < 0.001). Significant improvements were also observed in growth status, with 73.33% of children showing positive changes in nutritional classification. Specifically, 13.33% improved from underweight to slightly underweight, 20.00% from underweight to normal weight, and 40.00% from slightly underweight to normal weight (*p* < 0.001). Nevertheless, 23.33% showed no change, and 3.33% showed a decline in growth status (Table 3).

#### 3.2.2. Between-Group Comparisons (Experimental vs. Control Groups)

Mann–Whitney U tests revealed that, at post-intervention, the experimental group had significantly higher self-efficacy scores across all domains and overall than the control group (*p* < 0.01).

Similarly, caregiving behavior scores for food preparation, feeding, and care, as well as total caregiving behaviors, were significantly higher in the experimental group than in the control group (*p* < 0.001). However, no significant difference was observed between groups in food purchasing behaviors (*p* > 0.05).

The experimental group also showed significantly greater mean weight changes compared with the control group (*p* < 0.05) (Table 4).

## 4. Discussion

### 4.1. General and Health Characteristics of the Participants

The findings indicated that slightly more than half of the preschool children were male (51.67%). This distribution is consistent with national data in Thailand and previous studies suggesting that boys aged 1–5 years may be at greater risk of underweight than girls [22]. This increased vulnerability may be attributed to higher energy expenditure associated with greater physical activity during early childhood. Most children in the present study were aged 4–5 years, a developmental period marked by increased mobility, autonomy, and variability in dietary intake. These developmental changes may increase the risk of inadequate energy intake, particularly when feeding practices are inconsistent or nutritionally insufficient [16].

A considerable proportion of children did not consume three main meals per day (50.00%) and did not consume at least three servings of milk daily (85.00%). These behaviors are well-recognized risk factors for inadequate energy and nutrient intake and are consistent with findings from previous community-based studies [15]. Inadequate meal frequency and insufficient intake of nutrient-dense foods, such as milk, may contribute to poor weight gain and suboptimal growth.

Most primary caregivers were mothers or female caregivers (91.67%), reflecting traditional caregiving roles in Thailand and similar sociocultural contexts. Previous research has demonstrated that caregiver characteristics, including education and socioeconomic status, play an important role in shaping child nutrition and caregiving practices [13]. In the present study, many caregivers had secondary or lower levels of education, which may influence their knowledge, skills, and confidence in providing appropriate nutritional care. In addition, household income levels were relatively low, which may restrict access to a sufficient variety of nutritious foods. These findings are consistent with reports from the World Health Organization and prior studies indicating that undernutrition among children under five is more common in low-income households [3,22]. Nevertheless, most children lived with both parents, which may provide emotional and caregiving support that could positively influence child nutrition and development.

### 4.2. Effects of the Self-Efficacy Promotion Program Within the Experimental Group

Following the intervention, caregivers’ perceived self-efficacy increased significantly compared with baseline (*p* < 0.05). This improvement may be explained by the incorporation of Bandura’s four key sources of self-efficacy: mastery experience, vicarious experience, verbal persuasion, and emotional support [7]. Intervention components such as structured education sessions, observational learning, peer support, and ongoing follow-up through digital communication likely strengthened caregivers’ confidence in their ability to provide appropriate care. Higher self-efficacy has been associated with greater behavioral persistence, improved problem-solving, and more effective caregiving practices, all of which are essential for improving child nutritional outcomes.

Caregiving practice scores also improved significantly following the intervention (*p* < 0.05), indicating that increased caregiver confidence was accompanied by meaningful behavioral changes. According to social cognitive theory, individuals with higher self-efficacy are more likely to adopt and maintain health-promoting behaviors [7]. These findings are consistent with previous intervention studies showing that self-efficacy-based programs can improve caregiver practices and contribute to improved nutritional outcomes among young children [9,16,23].

Enhancing learning experiences through direct interaction with knowledgeable individuals and role models allows caregivers to observe successful caregiving behaviors, which may encourage positive expectations and behavioral imitation. This process can strengthen self-efficacy and support behavior change [7,8]. In the present study, learning from experienced caregivers and exchanging experiences helped improve caregivers’ understanding; however, caregiving challenges varied across families, and not all strategies could be directly applied to individual situations. Therefore, the researcher provided opportunities for emotional expression, one-on-one counseling, educational materials, and tailored guidance to build caregivers’ confidence in managing their child’s nutritional care. In addition, verbal encouragement and regular follow-up through the LINE application allowed caregivers to discuss problems, receive feedback, and feel encouraged when caregiving practices were successful [24]. This ongoing support promoted positive thinking, strengthened caregivers’ confidence, and contributed to more appropriate caregiving behaviors, which may support weight gain in preschool children [10].

Although the present study was not designed to determine the relative contribution of individual intervention components, ongoing follow-up and counseling through the LINE application may have played an important role in maintaining caregiver engagement throughout the intervention period. Continuous communication allowed caregivers to seek advice, receive feedback, and discuss challenges encountered in daily caregiving. In addition, group training sessions and role-model learning provided opportunities for observational learning and experience sharing, which are important mechanisms for strengthening self-efficacy according to Bandura’s theory. The combined effect of these components likely contributed to the observed improvements in caregiver self-efficacy, caregiving practices, and child weight outcomes.

However, no significant improvement was observed in food ingredient selection behaviors. This may be partly explained by relatively high baseline scores, suggesting that caregivers already had a basic level of knowledge in this area. In addition, external constraints such as limited household income may have restricted caregivers’ ability to modify food purchasing behaviors despite increased knowledge and motivation. In this study, the average monthly family income ranged from 10,800 to 11,480 THB, which was approximately 2.4–2.6 times lower than the average household income of Thai families (28,151 THB) reported by the National Statistical Office in 2025 [25]. Food accessibility and economic limitations may therefore have influenced caregivers’ decisions regarding food ingredient selection.

Importantly, children in the experimental group showed significant improvements in body weight following the intervention (*p* < 0.001). These findings suggest that enhancing caregiver self-efficacy and caregiving practices can lead to measurable improvements in children’s nutritional outcomes. Improved feeding practices, more consistent meal provision, and increased caregiver responsiveness may have contributed to improved energy intake and subsequent weight gain.

The magnitude of weight gain observed in the present study may differ from findings reported in previous studies because the intervention was implemented in community-based child development centers and targeted primary caregivers directly. Unlike clinic-based interventions, caregiver-focused programs may promote more sustained behavioral changes within the home environment, thereby influencing children’s daily dietary practices and nutritional intake.

Beyond statistical significance, the observed improvement in body weight may also be clinically meaningful for underweight preschool children. Even modest increases in body weight during early childhood can contribute to improved growth trajectories and reduce the risk of adverse health and developmental outcomes associated with undernutrition. From a public health perspective, interventions that enhance caregivers’ capacity to provide appropriate nutritional care may represent a practical and sustainable strategy for addressing childhood undernutrition in community settings, particularly in resource-limited areas.

According to Bandura’s Social Cognitive Theory, self-efficacy serves as a key determinant of behavior change. The findings suggest that improvements in caregivers’ self-efficacy may have enhanced their motivation, persistence, and ability to implement appropriate nutritional care practices, which subsequently contributed to improved weight outcomes among preschool children.

### 4.3. Comparison Between Experimental and Control Groups

Caregivers in the experimental group had significantly higher self-efficacy scores than those in the control group (*p* < 0.01), indicating the effectiveness of the intervention in enhancing caregivers’ confidence and caregiving capacity. The intervention incorporated key components of self-efficacy theory, including observational learning, verbal encouragement, emotional support, and continuous follow-up, which are known to strengthen behavioral capability and self-regulation [7]. These findings are consistent with previous studies demonstrating the effectiveness of self-efficacy-based interventions in improving caregiver behaviors and child nutrition outcomes [7,12].

Caregiving practice scores were also significantly higher in the experimental group compared with the control group (*p* < 0.05). This finding supports the theoretical pathway linking caregiver self-efficacy to behavioral change. Increased caregiver confidence may enhance motivation, consistency, and effectiveness in implementing appropriate feeding and caregiving practices. These findings are consistent with international recommendations emphasizing caregiver engagement as a key determinant of child nutritional outcomes [9].

Children in the experimental group showed significantly greater weight gain than the control group (*p* = 0.049) with a mean difference of 7.92 kgs. These findings indicate that the self-efficacy promotion program effectively enhanced caregivers’ confidence and caregiving practices, which may have contributed to the significant improvement in children’s body weight, suggesting a positive impact of the intervention on nutritional outcomes. Longer intervention and follow-up periods may be needed to observe more substantial changes in growth status.

Additionally, both groups continued to receive routine nutrition and health services provided by child development centers, which may have contributed to improvements in both groups and reduced the magnitude of between-group differences. Nevertheless, the greater improvements observed in the experimental group highlight the added value of self-efficacy-based interventions in strengthening caregiver behaviors and improving child nutritional outcomes.

In the study setting, caregivers may have increased awareness regarding appropriate nutrition; however, their ability to modify purchasing behavior may have remained limited due to financial constraints and the affordability of nutritious foods. In addition, access to diverse and nutritious food sources in the community may affect food purchasing choices, particularly in areas where healthy food options are limited or less accessible than commonly available alternatives.

Although food purchasing behavior among caregivers did not show a statistically significant change after the intervention, this finding should be interpreted within the local context of the study area. In community settings such as Nakhon Si Thammarat Province, caregivers’ food purchasing decisions are influenced not only by knowledge and attitudes regarding child nutrition, but also by structural and economic factors that may limit their ability to change purchasing practices.

Household income and financial constraints may play a major role in determining food choices, particularly among families with limited budgets. Even when caregivers have increased knowledge and awareness regarding appropriate nutrition, purchasing decisions may still depend on food affordability and the need to prioritize essential household expenses. In addition, accessibility to food markets and the availability of nutritious food in the local community may affect food purchasing behavior. Some families may have limited access to diverse food sources or may rely mainly on foods that are readily available and affordable in nearby areas.

Therefore, the lack of significant change in food purchasing behavior may not necessarily indicate limited effectiveness of the intervention program. Rather, it may reflect broader structural factors related to the local economic and social context that influence caregivers’ ability to apply nutritional knowledge in daily life. From a public health perspective, these findings suggest that improving child nutritional outcomes may require not only caregiver-focused interventions but also supportive community and structural measures, including improved access to affordable nutritious foods and economic support for families, to enable sustainable behavioral change.

## 5. Conclusions

Underweight among preschool children, a significant public health challenge that negatively impacts growth and development, is closely associated with family and caregiving practices. This study aimed to evaluate the effect of a self-efficacy development program for primary caregivers on caregiving behaviors and nutritional outcomes in underweight preschool children.

A quasi-experimental two-group pretest–posttest design was designed assigning 60 preschool children and their main caregivers to the experimental group exposed to the self-efficacy development program or control group receiving standard care. In addition, the experimental group’s scores of self-efficacy and behavior of caregiving improved more positively after intervention than the control group (*p* < 0.05). Food purchasing, food preparation, feeding practices and daily caregiving behaviors all saw improvements.

Furthermore, among preschool youngsters, a significant increase in mean body weight was reported for those in the intervention group after making baseline comparisons (*p* < 0.05), whilst no differences were recorded between groups to significantly affect children’s body weight. These findings indicate that the self-efficacy development program showed a significant improvement in caregivers’ caregiving capabilities and improvements in nutritional outcomes among underweight preschool children.

### 5.1. Strengths and Limitations of the Study

This study’s strength lies in the development of a structured 12-week self-efficacy enhancement program based on Bandura’s theory, combined with the use of the LINE application, which enabled convenient communication, continuous follow-up, emotional support, and health education for caregivers throughout the study. The program was practical and aligned with caregivers’ daily context, supporting its potential application in community child health promotion. However, this study has some limitations. Participant dropout occurred when caregivers were unable to attend group activities due to preschool absence, which reduced the sample size. Although communication through the LINE application helped maintain contact, participation in some activities remained limited. In addition, data were collected from child development centers in a single district, which may limit the generalizability of the findings. The follow-up period was also relatively short and may not fully reflect long-term changes in children’s nutritional outcomes.

### 5.2. Suggestions for Future Research

Future research should include longer intervention periods and follow-up assessments to evaluate the sustainability of caregivers’ self-efficacy, caregiving behaviors, and changes in preschool children’s nutritional outcomes over time. Studies in different settings are also recommended to enhance the generalizability of the findings. In addition, integrating family participation with digital technology or other health education media may improve access to information and support appropriate care for preschool children, particularly those at risk of undernutrition.

## Figures and Tables

**Table 1:** Comparison of general characteristics and health status of preschool children between the experimental and control groups (n = 60).

General Information and Health Status	Quantity (Percent)			t/X^2^	*p*-Value
	Experimental Group (n = 30)	Control Group (n = 30)	Total (n = 60)		
Sex				0.07 ^a^	0.79
Male	16 (53.33)	15 (50.00)	31 (51.67)		
Female	14 (46.67)	15 (50.00)	29 (48.33)		
Age (month)				1.24 ^b^	0.22
Mean (SD)	43.67 (7.09)	45.83 (6.39)			
Min–Max 95% CI Lower −5.65 Upper 1.32	30–60	30–60			
Children’s age group				2.15 ^a^	0.34
2.5 years–3 years	6 (20.00)	11 (36.67)	17 (28.33)		
>3–4 years	9 (30.00)	8 (26.67)	17 (28.33)		
>4–5 years	15 (50.00)	11 (36.67)	26 (43.33)		
Birth order				1.67 ^a^	0.19
1st	17 (56.67)	12 (40.00)	29 (48.33)		
>1st	13 (43.33)	18 (60.00)	31 (51.67)		
Dental health				0.27 ^a^	0.60
No tooth decay	16 (53.33)	18 (60.00)	34 (56.67)		
Tooth decay	14 (46.67)	12 (40.00)	26 (43.33)		
Eating the main meal				2.69 ^c^	0.24
<2 main meals/day	1 (3.33)	1 (3.33)	2 (3.33)		
2 main meals/day	11 (36.67)	17 (56.67)	28 (46.67)		
3 main meals/day	18 (60.00)	12 (40.00)	30 (50.00)		
Drinking milk				4.86 ^c^	0.14
1 carton of milk/day	6 (20.00)	12 (40.00)	18 (30.00)		
2 cartons of milk/day	20 (66.67)	13 (43.33)	33 (55.00)		
>2 cartons of milk/day	4 (13.33)	5 (16.67)	9 (15.00)		
Nutritional status				0.62 ^a^	0.43
Underweight (<−2 SD)	14 (46.67)	11 (36.67)	25 (41.67)		
Slightly underweight (−1.5–2 SD)	16 (53.33)	19 (63.33)	35 (58.33)		

^a^ Chi-square test; ^b^ Independent *t*-test; ^c^ Fisher’s exact test.

**Table 2:** Comparison of general characteristics of primary caregivers, self-efficacy scores and childcare behavior scores before the experiment between the experimental and control group (n = 60).

General Information	Quantity (Percent)			t	*p*-Value	95% Cl	
	Experimental Groups (n = 30)	Control Groups (n = 30)	Total (n = 60)			Lower	Upper
Sex				- ^c^	0.35		
Male	1 (3.33)	4 (13.33)	5 (8.33)				
Female	29 (96.67)	26 (86.67)	55 (91.67)				
Age (Years)				0.04 ^b^	0.97	−5.11	5.31
Mean (SD)	33.67 (10.33)	30.50 (9.81)					
Min–Max	22–57	22–58					
Age group				0.11 ^a^	0.74		
20–40 years	24 (80.00)	25 (83.33)	49 (81.67)				
41–60 years	6 (20.00)	5 (16.67)	11 (18.33)				
Education level				2.60 ^a^	0.27		
Primary education	6 (20.00)	9 (30.00)	15 (25.00)				
Secondary education	21 (70.00)	15 (50.00)	36 (60.00)				
Associate Degree/Vocational Certificate/Bachelor’s Degree	3 (10.00)	6 (20.00)	9 (15.00)				
Occupation				3.04 ^a^	0.22		
Employee	7 (23.33)	13 (43.33)	20 (33.33)				
Trade/Self-employed/Agriculture	13 (43.33)	8 (26.66)	21 (35.00)				
Housewife/Retired	10 (33.33)	9 (30.00)	19 (31.67)				
Monthly family income				0.10 ^b^	0.92	−1931.36	2131.36
Mean (SD) (Baht)	11,450.00 (4188.14)	11,350.00 (3651.05)					
Min–Max (Baht)	7500–20,000	7000–20,000					
<10,000 Baht	18 (60.00)	17 (56.67)	35 (58.33)	0.12 ^a^	0.94		
>10,000–15,000 Baht	5 (16.67)	6 (20.00)	11 (18.33)				
>15,000–20,000 Baht	7 (23.33)	7 (23.33)	14 (23.33)				
Family characteristics				1.07 ^a^	0.30		
Single family	17 (56.67)	13 (43.33)	30 (50.00)				
Extended family	13 (43.33)	17 (56.67)	30 (50.00)				
Marital status				2.22 ^a^	0.14		
Married	20 (66.67)	25 (83.33)	45 (75.00)				
Separated/Divorced/Widowed	10 (33.37)	5 (16.67)	15 (25.00)				
Primary caregiver				- ^c^	0.71		
Mother/Father	25 (83.33)	27 (90.00)	52 (86.67)				
Relative	5 (16.67)	3 (10.00)	8 (13.33)				
Average self-efficacy score before the experiment.	$\bar{x}$ (SD)	$\bar{x}$ (SD)					
Food purchasing	22.17 (2.74)	22.60 (1.92)		0.71 ^b^	0.48	−1.66	0.79
Food preparation	23.20 (3.46)	23.87 (2.75)		0.83 ^b^	0.41	−2.28	0.95
Feeding and care	40.17 (5.97)	38.30 (7.08)		1.10 ^b^	0.28	−1.52	5.25
Total self-efficacy score	85.53 (10.51)	84.77 (8.83)		0.31 ^b^	0.76	−4.25	5.78
Average score for childcare behavior before the experiment.	$\bar{x}$ (SD)	$\bar{x}$ (SD)					
Food purchasing behaviors.	37.57 (3.71)	37.70 (4.04)		0.13 ^b^	0.89	−2.14	1.87
Food preparation behaviors.	58.43 (7.68)	57.27 (8.46)		0.56 ^b^	0.58	−3.01	5.34
Feeding and care behaviors.	43.00 (6.43)	39.87 (7.76)		1.70 ^b^	0.09	−0.55	6.82
Total childcare behavior score	139.00 (14.21)	134.83 (17.95)		0.99 ^b^	0.32	−4.20	12.53

^a^ Chi-square test, ^b^ independent *t* test, ^c^ Fisher’s Exact Test.

**Table 3:** Comparison of primary caregivers’ self-efficacy scores, caregiving behavior scores, and preschool children’s weight and nutritional status before and after participation in the intervention program (n = 30).

Variable	Mean (SDSD)		Mean Rank	*Z*	*p*
	Before	After			
Primary caregivers’ self-efficacy					
Food purchasing	22.17 (2.74) (high level)	23.73 (1.87) (highest level)	11.17	3.62	<0.001
Food preparation	23.20 (3.46) (high level)	26.57 (3.49) (high level)	13.78	4.30	<0.001
Feeding and care	40.17 (5.97) (high level)	47.77 (6.31) (high level)	15.93	4.73	<0.001
Total self-efficacy scores of primary caregivers.	85.53 (10.51) (high level)	98.07 (10.79) (high level)	16.00	4.77	<0.001
Primary Caregivers’ behavior					
Food purchasing behaviors.	37.57 (3.71)	38.47 (2.42)	8.85	1.87	0.062
Food preparation behaviors	58.43 (7.68) (high level)	63.93 (6.27) (highest level)	15.02	3.59	<0.001
Feeding and care behaviors	43.00 (6.43) (high level)	49.43 (4.52) (high level)	15.08	4.31	<0.001
Total childcare behavior score of primary caregivers.	139.00 (14.21) (high level)	151.83 (11.39) (highest level)	16.04	4.32	<0.001
Average weight changes in preschool children 95% Cl (Before) Lower −0.02 Upper −0.03, (After) Lower 0.05 Upper 0.05	12.48 (1.65)	13.73 (1.92)	10.50	3.93	<0.001
Number of preschool children (Nutrition status)	Before (n = 30)	After (n = 30)		−4.31	<0.001
Number of children underweight.	14 (46.67)	5 (16.67)			
Number of children who were slightly underweight.	16 (53.33)	7 (23.33)			
Number of children with normal weight	0 (0.00)	18 (60.00)			

Wilcoxon signed-rank test was used for continuous variables.

**Table 4:** Comparison of primary caregivers’ self-efficacy, caregiving behaviors for preschool children and preschool children’s weight changes between the experimental and control group after the intervention.

Variable	Experimental Group		Control Group		*Z*	*p*
	Mean Rank	Consistent	Mean Rank	Consistent		
Primary caregivers’ self-efficacy						
Food purchasing	36.57	highest	24.43	highest	2.78	0.005
Food preparation	37.25	high	23.75	high	3.02	0.003
Feeding and care	40.92	high	20.08	moderate	4.63	<0.001
Total self-efficacy scores of primary caregivers.	40.60	high	20.40	high	4.49	<0.001
Primary Caregivers’ behavior						
Food purchasing behaviors.	33.02	highest	27.98	highest	1.18	0.237
Food preparation behaviors.	38.18	highest	22.82	high	3.48	0.001
Feeding and care behaviors.	41.43	high	19.57	high	4.86	<0.001
Total childcare behavior score of primary caregivers.	40.18	highest	20.82	high	4.30	<0.001
Average weight changes of preschool children	34.78		26.22		−1.97	0.049

Mann–Whitney U test.

## Data Availability

The original contributions presented in this study are included in the article. Further inquiries can be directed to the corresponding author.
